# Stress Biomarkers, Mood States, and Sleep during a Major Competition: “Success” and “Failure” Athlete's Profile of High-Level Swimmers

**DOI:** 10.3389/fphys.2016.00094

**Published:** 2016-03-14

**Authors:** Mounir Chennaoui, Clément Bougard, Catherine Drogou, Christophe Langrume, Christian Miller, Danielle Gomez-Merino, Frédéric Vergnoux

**Affiliations:** ^1^Fatigue and Vigilance Team, Neurosciences and Operational Constraints Department, French Armed Forces Biomedical Research InstituteBrétigny-sur-Orge, France; ^2^Sorbonne Paris Cité, EA 7330 VIFASOM Sommeil-Vigilance-Fatigue et Santé Publique, Université Paris DescartesParis, France; ^3^Team Lagardère, Scientific Expertise CenterParis, France

**Keywords:** cortisol, α-amylase, chromogranin-A, stress, mood, sleep

## Abstract

The aim of this study was to evaluate stress markers, mood states, and sleep indicators in high-level swimmers during a major 7-days competition according to the outcomes. Nine swimmers [six men and three women (age: 22 ± 2 and 22 ± 4 years, respectively)] were examined. Before (PRE) and after (POST) each race (series, semi-finals, and finals), salivary concentrations of cortisol, α-amylase (sAA), and chromogranin-A (CgA) were determined. Mood states were assessed by the profile of mood state (POMS) questionnaire completed before and after the 7-days, and self-reported sleep diaries were completed daily. In the “failure” group, cortisol and sAA significantly increased between PRE-POST measurements (*p* < 0.05), while sCgA was not changed. Significant overall decrease of cortisol (-52.6%) and increase of sAA (+68.7%) was shown in the “failure group.” In this group, fatigue, confusion and depression scores, and sleep duration before the finals increased. The results in the “success” group show tendencies for increased cortisol and sCgA concentrations in response to competition, while sAA was not changed. Cortisol levels before the semi-finals and finals and sCgA levels before the finals were positively correlated to the fatigue score in the “failure” group only (*r* = 0.89). sAA levels before and after the semi-finals were negatively correlated to sleep duration measured in the subsequent night (*r* = −0.90). In conclusion, the stress of the competition could trigger a negative mood profile and sleep disturbance which correspond to different responses of biomarkers related to the hypothalamo-pituitary-adrenal axis and the sympathetic nervous system (SNS) activity, cortisol, sAA, and CgA.

## Introduction

High-level swimmers, involved in different specialties (100, 200 m, stroke, butterfly…), have to compete on several days during major events such as national championships. Regularly, series and semi-finals are programmed on the same day, in the morning and the afternoon respectively, while the finals are planned on the next day afternoon. During these major competitions, physiological influences of stress, mood states, and sleep may interact and rebound effects on performance may be observable. Sport competition is well known to induce a stress response, which can even appear in anticipation of the event (Kraemer et al., 2001; Kivlighan et al., 2005; Diaz et al., 2013). This stress response is regulated through the activation of the hypothalamus-pituitary-adrenal (HPA) axis and sympathetic nervous system (SNS; Chrousos and Gold, 1992; Chrousos, 2009), which intervene concomitantly. Saliva being a useful, non-invasive alternative to the collection of serum and plasma, it rapidly develops as a tool for the assessment of the stress response on the sports field (Papacosta and Nassis, 2011). Since both HPA axis and SNS activity can be reflected by changes in salivary concentrations of proteins such as cortisol, alpha-amylase, and chromogranin A, several studies investigated their changes during high-level competitions (Kivlighan and Granger, 2006; Filaire et al., 2009a; Azarbayjani et al., 2011).

Cortisol is a steroid hormone, secreted from the adrenal cortex via the HPA axis, which increases in response to stressors including physical exertion (Gallacher and Petersen, 1983; Papacosta and Nassis, 2011). Increased cortisol levels are not only related to anxiety or depression but also to high-intensity exercise, with proportional elevation according to exercise intensity. Cortisol is considered the main hormone responsible for catabolic processes, as it reduces protein synthesis, increases protein degradation and inhibits the inflammatory process and immunity. Salivary cortisol can reliably reflect the HPA activity and represents plasma and/or total cortisol concentration in response to exercise (Filaire et al., 2009a; Obayashi, 2013). Alpha-amylase is one of the major salivary enzymes in humans, and is secreted from the salivary glands in response to sympathetic stimuli. Its concentration in saliva reflects blood levels of catecholamines, particularly norepinephrine (Chatterton et al., 1996), and thereby, salivary α-amylase (sAA) is considered as a useful tool for evaluating the SNS activity (Walsh et al., 1999). This protein is highly sensitive to stress, and its changes are more remarkable than those in salivary cortisol after the same mental stress event (Obayashi, 2013). Nonetheless, both of the two biological stress-related markers, cortisol and sAA, do not necessarily correlate (Chatterton et al., 1996). It is likely the exact nature of the coordination between the HPA axis and the SNS system (additive or interactive; opposing or complementary) is not clearly elucidated (Kivlighan and Granger, 2006). Chromogranin-A (CgA), which is an acidic glucoprotein that is released along with catecholamines from the adrenal medulla and the sympathetic nerve endings (O'Connor et al., 1984), is also perceived as a novel stress marker, notably psychological stress. Salivary CgA (sCgA) is produced by submandibular glands and secreted into saliva by stimulation with noradrenaline and acetylcholine (Saruta et al., 2005). In stressful situations such a car driving on an expressway or public speaking, sCgA levels are significantly increased, before an elevation of salivary cortisol concentrations (Nakane et al., 1998, 2002). Moreover, in the context of sport sciences, since increased sCgA levels were observed after high-intensity exercise such as incremental maximal swimming test, sCgA has also been proposed as a reliable cardiovascular marker (Gallina et al., 2011; Bocanegra et al., 2012; Toda et al., 2013).

High-level competitors are directly and continuously exposed to both physiological and psychological stressors (Salvador and Costa, 2009; Costa and Salvador, 2012), which can interact and affect performance. The profile of mood states questionnaire (POMS; McNair et al., 1971) is a relevant tool to study adaptation/deadaptation to training. It is noteworthy that despite the POMS scores may precisely reflect changes in athletes' mood states and can be a useful tool to predict the chance of success or failure during a competition, the relationship between biological variables and mood states is not clearly identified (Gatti and De Palo, 2011). In a recent study, while combining psychological and biological variables to more accurately predict performance during a swimming competition, some authors failed to evidence any correlation between the cortisol awakening response and mood states (Diaz et al., 2013).

Sport performances have been shown to be dependent upon both quality and quantity of sleep that has been taken before a competition (Halson, 2014; Chennaoui et al., 2015). Either total or partial sleep deprivation has been shown to impair vigilance and sustained attention and to alter hormonal processes in healthy young men (Arnal et al., 2015) and to decrease short-term maximal performances of judo competitors (Souissi et al., 2013). Conversely, exercise by itself is known to have an impact on subsequent sleep: it contributes to well-being and improved mood states which contribute to a better sleep quality (Souissi et al., 2012; Chennaoui et al., 2015). Frequently, physical exercise is associated with reduced sleep latencies and fragmentation index, but also to an increased deep slow wave sleep. Nonetheless, in the case of an acute and strenuous exercise such as a major competition, stress caused by the uncertainty of the results and nervousness may lead to poor sleep quality and quantity (Juliff et al., 2015).

The aim of this study was to evaluate changes in stress biomarkers, self-reported mood states and sleep in relation to the outcomes of a major competition while distinguishing “success” and “failure” athlete's profile of high-level swimmers. Based on literature data (Kivlighan and Granger, 2006), we hypothesized that athletes who perceived the less negative stress related to biomarkers would be in better global mood states and sleep throughout the competition, allowing them to benefit from a good recovery and thus would have more success during the competition.

## Materials and methods

### Subjects

Nine professional high-level swimmers [six men (age: 22 ± 2 years old, height: 188.62 ± 6.84 cm, weight: 82.25 ± 7.04 Kg); three women (age: 22 ± 4 years old, height: 167.67 ± 5.51 cm, weight: 60.33 ± 3.78 Kg)] voluntarily took part in this study. All participants were Elite athletes, members of the Lagardère Paris Racing team and competing at the international level during the year of the experiment. The sample included two Olympic medalists, one World championship medalist, one European championship medalist, two Olympic finalists, one European champion, and one French Champion. All were ranked between the 80th and the 1st place at the Fédération Internationale de Natation (FINA) at the moment of the study. The training volume of swimmers during the three weeks before competition was successively 65, 55, and 45 km/week, then it was reduced to 40 km/week during the competition week. During the anterior mesocycles, the training volume was 85 km/week including a mean of 85% aerobic and 15% anaerobic (Chamari and Padulo, 2015).

Written informed consent was obtained from all participants before starting the experiment. The study was approved by the medical committee of the French swimming federation, according to the guidelines of the ethical committee of the University of Paris V Cochin (Paris, France), and was conducted in respect to the ethical standards of the Declaration of Helsinki.

All athletes were in good health, were in regular training for the past 3 years, were not using medications before or during this study, and had no history of psychiatric, somatic, or sleep disorders. They all identified themselves as non-smokers. Their general health was evaluated by a sport physician, and female participants reported to be regularly menstruating.

### Procedures

The present study was framed within a wider project which involved following up the subjects throughout the sports training season in order to analyze their psychophysiological adaptation during this period. To evaluate the influence of the variables on a major competition and its outcome, the following assessment was carried out during the French national swimming championship. This competition was particularly important for these athletes as it represented the opportunity to realize minima cut-offs, qualifying them for the European swimming championships.

The athletes completed a psychological assessment for evaluating their mood states on the first (Monday) and the last mornings (Sunday) of the week of competition, at the same time-of-day (09:00 h). In addition, to identify precisely the changes occurring on stress markers throughout the competition, saliva samples were collected just before and after the different races (series, semi-finals, finals) for cortisol, α-amylase, and chromogranin-A determination. Sleep parameters were estimated during the whole week of the competition throughout actigraphy measurements and the completion of a sleep diary.

Swimming competitions are organized according to an elimination process (edited by the FINA, regarding the use of eight lanes) in which participants of the series, then half-finals are qualified for the next step, so as to oppose eight athletes during the finals. As only eight athletes are opposed in the final race, we considered the athletes who finished in the first four places of their finals as representing the “success” athlete's profile (*n* = 4). In contrast, 5 of the 9 athletes finished over the 4th place of their finals and were considered as the “failure” athlete's profile.

### Measurements

#### Biological variables

Before saliva collection, subjects were required to rinse out their mouths for 1 min with water to remove any substances that may affect cortisol, α-amylase, and chromogranin-A levels. To standardize saliva collection, subjects were seated in comfortable position, with slightly lowered head, allowing spontaneous saliva flow in the mouth. Chewing gum or mints, and teeth brushing were prohibited for at least 1 h before sampling. Subjects were instructed to swallow to empty the mouth before unstimulated whole saliva sample was collected. Once collected, saliva samples were immediately placed on ice, transported to the laboratory, and stored frozen (−20°C) until assayed. All saliva samples were subject to a single freeze thaw cycle. On the day of assay, samples were centrifuged (10,000 × g, 3 min) to remove particulate matter, and clear samples were transferred into appropriate test wells.

The salivary levels of cortisol, α-amylase, and chromogranin-A were assessed by commercial kits (Salimetrics, State college, USA and Yanaihara Institut Inc., Shizukoa, Japan). For singlet determinations, the test used 25 μl of saliva for cortisol, 10 μl of saliva for α-amylase, and 50 μl of saliva chromogranin-A. Assays were made in duplicate and intra- and inter-assay coefficients of variations (CVs) were 3.0 and 3.0% for cortisol, 6.7 and 3.6% for α-amylase, 8.2 and 12.4% for chromogranin-A, respectively. Salivary cortisol data are expressed in nanomoles per liter (nmol.L^−1^). Salivary α-amylase data are expressed in Unit per microliter (U.mL^−1^). Salivary chromogranin-A data are expressed in picomoles per milliliter (pmol.mL^−1^). The analytical range of sensitivity for cortisol, α-amylase, and chromogranin-A was 0.33-83 nmol.L^−1^, 2-400 U.mL^−1^, and 0.14-33 pmol.mL^−1^, respectively.

Raw concentrations obtained from before and after each race samples were considered for statistical analysis. Moreover, the difference between before and after each race measurements was calculated to estimate the exercise-induced stress response.

#### Mood states assessment

The POMS-f (French version) is a series of 65-adjectives measuring six mood states (Tension, Depression, Anger, Vigor, Fatigue, Confusion) on a five-point Likert scale from 0 (not at all) to 4 (extreme), in relation to the context (Cayrou et al., 2000). The test takes approximately 3 min to administer in a paper and pencil form. The subjects were asked to state how they felt at the moment. The Total Mood Disturbance [(Tension + Depression + Anger + Fatigue + Confusion) – Vigor] was calculated. The raw scores for each item were analyzed to further investigate changes in mood states.

#### Sleep assessment

Sleep efficiency was calculated by means of actigraphy measurements and sleep diaries (Halson, 2014) completed during the whole week of the competition (six nights). The Sleep Efficiency Index (SEI) is considered as the ratio between Total Sleep Time (TST) and Time In Bed (TIB). The TST is determined by: Sleep Period Time (SPT: time between sleep and awakening) – Sleep Onset Latency (SOL) – Wakefulness After Sleep Onset (WASO). We only focused on the night preceding the different races.

### Statistical analyses

Salivary cortisol, alpha-amylase, and chromogranin-A concentrations were analyzed using a Friedman ANOVA including the different samples (series before, after, difference before/after; semi-finals before, after, difference before/after; finals before, after, difference before/after) which were realized for each group separately (success, failure). Sleep parameters were analyzed using a Friedman ANOVA including the different nights preceding the races (series, semi-finals, finals) measured for each group separately (success, failure). In case of significance, the effect size was estimated by the Kendall's coefficient of concordance and a Wilcoxon test was performed to evidence pairwise differences. POMS scores recorded for each item (Tension, Depression, Anger, Vigor, Fatigue, Confusion) before and after the competition were analyzed using a Wilcoxon test for pairwise comparisons, for each group separately (success, failure). To further evidence differences between success and failure athlete's profiles, Mann-Whitney comparisons were performed for each variable.

To examine the relationship between salivary cortisol, sAA, and sCgA concentrations recorded before each race, mood states observed before the competition and sleep parameters observed for the night preceding each race, the Spearman's rank correlation coefficient was used.

Results are described as means ± SEM for each assessment. All statistical differences were considered as significant for a *p*-value < 0.05. All statistical analysis were performed using Statistica software® v.10 (Statsoft Inc., France).

## Results

### Biological variables

**Figure 1:**
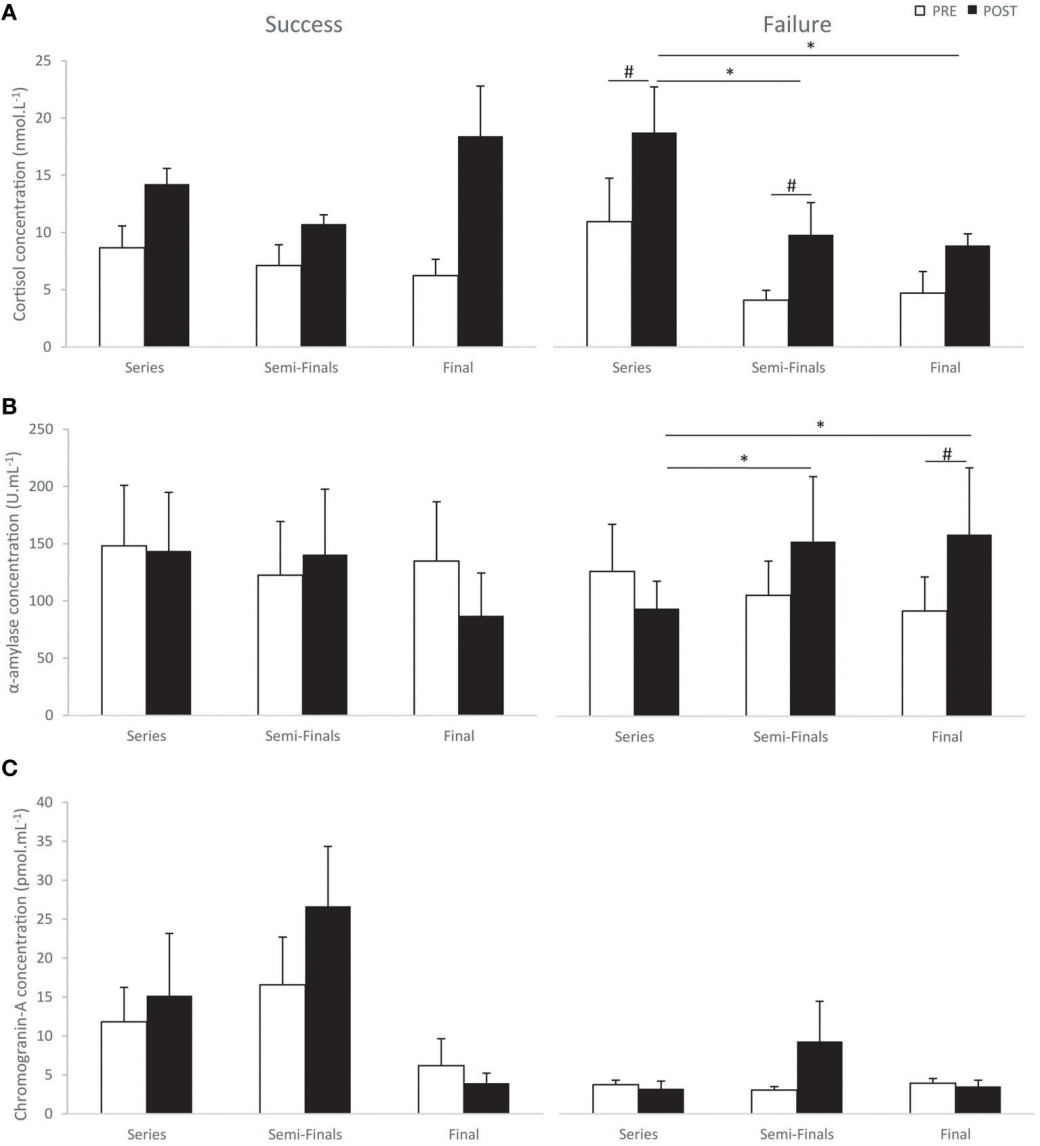
**Cortisol (A); Alpha-amylase (B); Chromogranin-A (C) concentrations before (PRE) and after (POST) series, semi-finals and finals for the “success” (left) and “failure” groups (right) (means ± SEM)**. ^#^significant difference between PRE and POST (*p* < 0.05); and ^*^significant difference between series, semi-finals, and final races (*p* < 0.05).

The results for stress evaluation indicated that cortisol concentrations were significantly influenced by the different races for the “failure” group (χ^2^ = 17.57; Kendall's *W* = 0.70; *p* < 0.01). Concentrations after (POST) the series were significantly higher than before (PRE) the series and also higher than after the semi-finals and finals. Concentrations after the semi-finals were also higher than before this race (Figure 1A). Cortisol levels were also significantly influenced by the different races for the “success” group (χ ^2^ = 14.86; Kendall's *W* = 0.74; *p* < 0.05). However, pairwise comparisons only indicated that measurements realized before the series and finals tended to be lower than after each of these races respectively, for the “success” group (*p* = 0.06). Mann-Withney comparisons indicated that cortisol levels after the finals tended to be higher for the “success” than the “failure” group (*Z* = 1.83; *p* = 0.06).

While comparing the increase between before and after each race measurements, it is interesting to note that it reached significance only for the “success” group (χ^2^ = 6.50; Kendall's *W* = 0.81; *p* < 0.01). More precisely, this increase tended to be higher for the finals than for the series and semi-finals (*p* = 0.06). Mann-Whitney comparisons indicated that the increase of cortisol concentrations during the finals was significantly higher for the “success” group than for the “failure” group (*Z* = 2.08; *p* < 0.05).

Alpha-amylase concentrations were significantly different between measurements for the “failure” group only (χ^2^ = 12.64; Kendall's *W* = 0.50; *p* < 0.05). Measurements realized after the series were lower than after the semi-finals and finals (Figure 1B). Salivary α-amylase levels after the finals were significantly higher than before this race. Furthermore, this increase observed in the “failure” group, only during the finals was significantly higher than in the “success” group (*Z* = −2.32; *p* < 0.01).

Chromogranin-A concentrations were significantly different between measurements for the “success” group only (χ^2^ = 13.43; Kendall's *W* = 0.67; *p* < 0.05). Nevertheless, only tendencies were reported by Wilcoxon tests while comparing pairs of data. Chromogranin-A concentrations after the semi-finals tended to be higher than after the series and finals (*p* = 0.06; Figure 1C). Concentrations before the semi-finals also tended to be higher than before the finals for the “success” group (*p* = 0.06). Mann-Whitney comparisons indicated that Chromogranin-A concentrations before the semi-finals were higher for the “success” group than for the “failure” group (*Z* = −1.96; *p* < 0.05).

### Mood states assessment

**Figure 2:**
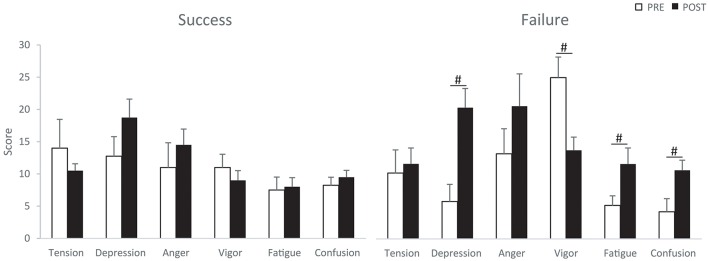
**POMS scores for the “success” (left) and “failure” groups (right) at the beginning (PRE) and end (POST) of the competition (means ± SEM)**. ^#^significant difference between PRE and POST (*p* < 0.05).

The Total Mood Disturbance index significantly increased in the “failure” group (*Z* = 2.02; *p* < 0.05). Mann-Whitney comparisons indicated that this increase in TMD index (13.6 points vs. 61.0 points) represented a 4.5 fold change which was significantly higher than for the “success” group (*Z* = −2.33; *p* < 0.05). The raw POMS sub-scores indicated significant changes after the competition for the “failure” group (χ^2^ = 40.08; Kendall's *W* = 0.80; *p* < 0.001). More precisely, depression, fatigue and confusion scores increased after the competition, while vigor score decreased in this group (Figure 2). POMS scores were also significantly influenced by the competition for the “success” group (χ^2^ = 20.14; Kendall's *W* = 0.50; *p* < 0.05). However, pairwise comparisons only indicated that depression and anger scores measured after the competition tended to increase in comparison with the beginning of the competition. Surprisingly, Mann-Whitney comparisons indicated that the “success” group had lower vigor scores than the “failure” group before the competition (*Z* = −2.21; *p* < 0.05).

### Sleep assessment

Considering sleep evaluation, sleep duration was significantly affected during the competition for the “failure” group (χ^2^ = 9.58; Kendall's *W* = 0.96; *p* < 0.01). Sleep duration was longer the night before the finals in comparison with the night preceding the series and semi-finals (Table 1). Mann-Whitney comparisons indicated that the “failure” group had longer sleep duration than the “success” group before the finals (*Z* = −1.96; *p* < 0.05). No significant difference was observed for SEI, regardless of the group.

**Table 1:** **Sleep duration and Sleep Efficiency Index (SEI) for the nights preceding the series, semi-finals, and final races for the “success” and “failure” groups**.

		**Series**	**Semi-finals**	**Finals**
Sleep duration	“success” group	8.75 (0.57)	8.62 (0.33)	8.75 (0.57)
	“failure” groups	8.69 (0.37)	8.19 (0.21)	10.55 (0.25)^*^, ^$^
SEI	“success” group	91.20 (3.69)	85.62 (4.32)	84.80 (8.36)
	“failure” groups	90.18 (2.59)	91.16 (2.61)	92.43 (1.17)

Values are Means (SEM);

* significantly different from series;

$ *significantly different from semi-finals (p < 0.05)*.

### Association between biological variables and mood

In the “failure” group, cortisol levels measured before the series and semi-finals were positively correlated with vigor (*r* = 0.90). Our results also showed that the higher the cortisol levels before the semi-finals and finals, the higher fatigue score (*r* = 0.89). Fatigue score was also higher when sCgA concentration was higher before the finals, in the “failure” group only (*r* = 0.89). The stress response (pre-post measurements) observed for cortisol during the series was positively correlated with depression (*r* = 0.90), while this difference during the finals was negatively correlated to confusion (*r* = −0.95).

### Association between biological variables and sleep

In the “failure” group, sleep duration observed in the night preceding the semi-finals was negatively correlated to the stress response (pre-post measurements) for cortisol in the semi-finals (*r* = −0.97). Sleep duration measured in the night preceding the finals (following the semi-finals) was negatively correlated to sAA levels before and after the semi-finals (*r* = −0.90). Moreover, the sleep duration of this particular night was positively (*r* = 0.90) and negatively (*r* = −0.90) correlated to the stress response (pre-post measurements) for CgA and sAA during the finals, respectively.

Sleep efficiency measured in the night preceding the series was negatively correlated (*r* = −0.90) to the stress response (pre-post measurements) for CgA during this race. Sleep efficiency in the night following the semi-finals was negatively correlated to cortisol concentration before the semi-finals (*r* = −0.90).

### Association between mood and sleep

In the “failure” group, confusion was negatively correlated to sleep duration before the series (*r* = −0.97). Depression was also negatively correlated to sleep duration before the semi-finals (*r* = −0.97).

## Discussion

This study is the first to put into relationship salivary concentrations of cortisol, α-amylase, and chromogranin-A, mood disturbances and sleep indicators in elite athletes during a national swimming competition, while differentiating “success” and “failure” athlete's profile. Our major findings indicate these biomarkers of stress evolved differently according to the outcome of the competition, which was also reflected by particular changes in mood states and sleep parameters. In the “failure” group, the regulation of the stress response is operated by the interaction between cortisol concentrations which decreased in response to competition from the series to finals, while on the opposite, sAA concentrations increased. No significant change was observed in sCgA levels in this group. The changes in biological variables may be linked to psychological aspects in the “failure” group, as mood states and sleep indicators were also modified.

In our study, the Total Mood Disturbance index significantly increased in the “failure” group only, reflecting an increase in worse mood states (Rietjens et al., 2005). In fact, a number of negative items (Fatigue, Depression, Confusion) were increased in this group after the competition, while Vigor decreased. These observations indicated that despite a rough investment in the competition, the poor results of the “failure” group' athletes affected their psychological state. If results are analyzed with caution, the athletes of the “Success” group presented “Depression” and “Confusion” scores that also tended to be higher at the issue of the competition. This event was chosen because it was an opportunity for the athletes to realize the minima cut-offs, giving them the opportunity to qualify for the European championship. While successful athletes realized a good performance in the finals, this was not sufficient enough to qualify them for the European championship, which tends to increase negative items in the “success” group.

While the “failure” group seemed to be fatigued throughout the competition as attested by the evolution of the POMS negative items, they increased their sleep duration before the finals. This increase in sleep duration attested the need for these athletes for an additional recovery, which failed to be sufficient to perform in the finals. In contrast with the “failure” group, sleep duration and sleep efficiency of the “success” group did not change throughout the days of competition. These observations may suggest that a good sleep hygiene and regular sleep characteristics are determinant in order to perform (Chennaoui et al., 2015).

Cortisol levels were not significantly different between both groups before each race. This confirms that pre-competition cortisol, which is a major mediator of the organism's response to a challenging situation would have little, if no effect, on these high-level athletes (Zilioli and Watson, 2012). Our results also show for the “failure” group a significant increase in pre/post competition measurements in series and semi-finals, but not in the finals, while the overall cortisol level decreased throughout the competition. In a previous study, a subsequent decrease of cortisol level after repeated maximal exercise associated with an acute state of fatigue was also observed (Viru et al., 2001). In contrast, the greatest responsiveness (2.9 fold) was observed during the finals for the “success” group, which was significantly higher than for the “failure” group. These results are in accordance with those of previous studies reporting that higher end-competition cortisol concentrations were associated with better performance (Snegovskaya and Viru, 1993; Passelergue et al., 1995; Le Panse et al., 2012). The exercise-induced increase in circulating cortisol concentration is essential for the normal metabolic response to exercise and in the process of adaptation to repeated bouts of demanding exercise (Viru and Viru, 2004). Thus, it seems that the athletes from the “success” group had a greater adaptation to competition than the athletes from the “failure” group. In our study, correlations between cortisol responses and the self-reported psychological response in the POMS questionnaire and daily sleep characteristics were significant in the “failure” group only. As previously observed, cortisol concentrations before semi-finals and finals were positively correlated to the fatigue score (Viru et al., 2001; Viru and Viru, 2004). Moreover, cortisol concentrations before the semi-finals were also negatively correlated to sleep efficiency in the following night (before the finals). Our results showed that sleep duration was significantly higher before the finals in the “failure group.” This increase in sleep duration was positively associated to higher sleep efficiency. In addition, the cortisol response in the semi-finals was negatively correlated to the sleep duration during the night preceding this race. All of these results suggested that high cortisol concentrations are associated with short sleep duration and low sleep efficiency in the following night, and this could be detrimental for performance. Sleep is crucial for a good recovery and for the realization of an optimal performance (Chennaoui et al., 2015).

Salivary α-amylase did not show any significant fluctuation during the competition in the “success” group. In addition, no significant changes were noticed in the “failure” group in pre competition measurements. These observations confirmed that sAA changes in response but not in anticipation of competition (Kivlighan and Granger, 2006). Salivary α-amylase increased in the “failure” group throughout the competition in an opposite way compared to cortisol levels. This mirror evolution is such that pre/post competition measurements became significant only when cortisol measurements did not, more precisely during the finals. This increase in sAA response was 1.7 fold during the finals, while no significant change was observed in the “success” group. Similar results indicating a two-fold increase in sAA induced by psychological stress were previously reported (Bosch et al., 1996). A 1.4 fold increase in sAA was also shown while playing a video game (Skosnik et al., 2000). In response to physical intense exercise, sometimes associated to the psychological stress of the competition, increased sAA concentration was mainly described (Kivlighan and Granger, 2006; de Oliveira et al., 2010; Chiodo et al., 2011; Gallina et al., 2011). However, we can only confirm these previous results in the “failure group,” which is in contrast with a previous study reporting that greater levels of sAA were associated with the fastest race time (Kivlighan and Granger, 2006). These authors suggested that the increase in sAA levels could be linked to the manageable aspect of the situation, as an increase SNS activity may be specifically related to the mobilization effort in situations perceived as controllable (Henry, 1992). Nonetheless, our sample was composed of high-level swimmers and their tough experience of the competition may have led to an equal confidence in their own capabilities (as attested in the POMS scores observed before the competition). Since no difference between both groups was observed in the overall levels of sAA throughout the competition, it seems less hazardous to focus on its primary role. Salivary α-amylase is meant to begin digestion of complex starches, sugars, and carbohydrates (Lebenthal, 1987). As a consequence, we suggest that an increase in sAA levels in the “failure group” may underline a higher SNS activity for restoration of energy reserves when the HPA activity is low, i.e., sAA increases at the finals whereas cortisol concentration did not. In addition, the correlation analysis showed in the “failure group” that sAA concentrations before and after the semi-finals were negatively correlated to sleep duration in the following night (before the finals). The sAA response during the finals was negatively correlated to sleep duration observed in the preceding night. This suggests a relationship between the increased SNS activity reflected by sAA response and lowered HPA activity related to cortisol response during a stress period with fatigue and disturbed sleep.

The present study showed in the “success” group that sCgA concentration after semi-finals tended to be higher than after the series and after the finals. These higher concentrations were also significantly higher than in the “failure” group. Some studies identified both sCgA and sAA as potential biomarkers of sympathetic activity for evaluating the relationship between SNS activity and mucosal immunity following psychological and physical stress in normal and pathological conditions (Takatsuji et al., 2008; Bocanegra et al., 2012; Obayashi, 2013). Both these types of stressors stimulate the salivary glands to increase the secretion of sAA, while sCgA has been shown to increase immediately in response to an acute academic examination stress (Takatsuji et al., 2008). Furthermore, other studies also linked sCgA response to mental health status and particularly depression. In university students with a high score for depression in the mental health status questionnaire [GHQ (General health)-28], sCgA awakening response was decreased via a chronic-stress related attenuation of the SNS activity (Den et al., 2011). In the same way, using the GHQ-28 questionnaire, it has been previously described that only the low-score group showed significant increase in CgA concentration (Toda et al., 2007). In his review, Obayashi (2013) stated that from a durability point of view, salivary CgA may be the most useful biochemical marker of chronic mental stress. Nonetheless, few data are available regarding the relationship between sCgA and exercise. Salivary CgA levels appear to increase in proportion to the increase in plasma noradrenaline levels during a brief exercise period (Chatterton et al., 1996), and at the end of a short-duration physical test to exhaustion (Gallina et al., 2011). In the latter study, sCgA correlates with both the cardiovascular response to physical exercise and the subjective perception of exercise intensity while no correlation was detected between sAA and these indices of exercise intensity. In our study, correlations between sCgA responses and mood scores were observed in the “failure group” only: sCgA concentrations before the finals were positively correlated to fatigue assessed at the beginning of the competition. In addition, sCgA response in the finals was positively correlated to sleep duration in the previous night. Thus, we can see that cortisol and sCgA concentrations before the finals were associated positively with fatigue felt by the less competitive swimmers.

Taken together, our results confirm that the stress response to a major competition is regulated via two major mechanisms. There is at first, a short latency catecholamine component, depending upon the SNS, and then, a slower acting glucocorticoid response, depending upon HPA activation. Our results tend to precise that both these mechanisms are acting in a combined but opposite way, HPA activity probably being compensated by even CgA or AA release, depending on the athlete's profile. More precisely, our results showed that among elite international swimmers, those who succeeded had higher sCgA concentrations without sAA increase at post-race, which may be related to the absence of changes in negative mood states and sleep duration. All of these psychobiological advantages may have facilitated recovery between races and contributed to the final success in the competition. In contrast, the increase in negative mood states and fatigue induced longer sleep duration before the finals in athletes who failed, and the increase in sAA release should compensate cortisol exhaustion along the competition in order to maintain energy availability.

### Limitations of the study

This study's multiple approaches was challenging and allowed new insights into stress, mood states and sleep influences on a swimming competition' outcomes. However, this research presents limitations including sample size, gender difference and the influence of saliva collection timing on measured concentrations. The number of athletes is limited because of the competition level but it presents the advantage to benefit from very homogeneous sample for the age range, with similar and very high level of performance in addition to extensive experience in professional competition. We can also rule out a possible influence of gender, since no differences on cortisol, sAA, and sCgA were previously observed at baseline and in response to a lecture stress among 52 young participants (Filaire et al., 2009b). Regarding saliva collection timing, it was adjusted to each race: series were always performed in the morning, while semi-finals and finals were realized in the afternoon. Hormonal concentrations are known to be influenced by circadian rhythmicity. Cortisol has been shown to peak within the first 30 min after awakening and then decrease gradually throughout the day until bedtime. In contrast, sAA concentrations show lowest levels after awakening and highest levels in the evening (Rohleder and Nater, 2009). Nonetheless, it has been shown that sAA similarly increases in morning and afternoon exercises (Li and Gleeson, 2004) and good stability of sAA measured between +1 and +11 h after awakening has been reported (*r*'s between 0.48 and 0.65, all *p*'s < 0.01) (Wolf et al., 2008). Chromogranin-A levels peak upon awakening and then quickly decreased to the nadir after 1 h, remain low during the day and increase again late at night (Den et al., 2007). Even if circadian influences cannot be neglected, since all the measurements carried out in the morning were realized between 10:00 and 11:45 for the series and between 14:30 and 16:30 for the semi-finals and finals, results may be more representative of exercise-induced changes than diurnal rhythmicity influence.

### Practical applications

To better prepare high-level athletes to cope with the stress induced by a competition, in addition to the POMS, other questionnaires evaluating anxiety/depression (State-Trait Anxiety Inventory; Recovery Stress Questionnaire for athletes) may be useful to better discern the athlete' psychological profile (Durguerian et al., 2015). Data from our study tend to show that successful athletes present higher concentration of sCgA. These results deserve to be confirmed in a larger number of athletes as it may provide a useful prognostic information on coping styles. Based on analysis, our results also suggest that in long-term follow-up, sCgA and sAA could be used in addition to other stress markers such as cortisol and testosterone, allowing the evaluation and prevention of fatigue (Guilhem et al., 2015). Associated to sleep indicators analysis, this psychophysiological assessment of athletes will give the opportunity to identify how an athlete cope with stress induced by a competition and various mental preparation strategies should be advised for performance enhancement. The effectiveness of these interventions may be evaluated in low-level competitions. Finally, our results do not only have implications for sport practitioners. Salivary α-amylase and sCgA can be considered as reliable and convenient indicators of the adrenergic response to exercise bouts (Bocanegra et al., 2012). In subjects affected by arterial hypertension or cardiovascular disease, the noninvasive analysis of these stress biomarkers could be useful to adjust the intensity of physical exercises during rehabilitation programs (Gallina et al., 2011).

## Author contributions

Conception or design of the work: MC, CM, FV. Acquisition, analysis, or interpretation of data for the work: MC, CB, CD, DG, CL. Drafting the work or revisiting it critically for important intellectual content: MC, CB, CD, DG. Final approval of the version to be published: MC, CB, CD, DG, CL, CM, FV. Agreement to be accountable for all aspects of the work in ensuring that questions related to the accuracy or integrity of any part of the work are appropriately investigated and resolved: MC, CB, CD, DG, CL, CM, FV.

## Funding

Team Lagardere SNC funding.

### Conflict of interest statement

The authors declare that the research was conducted in the absence of any commercial or financial relationships that could be construed as a potential conflict of interest.
